# Ecological Risk Assessment of Organochlorine Pesticides and Polychlorinated Biphenyls in Coastal Sediments in China

**DOI:** 10.3390/toxics12020114

**Published:** 2024-01-29

**Authors:** Jie Wang, Qi Zhao, Fu Gao, Ziye Wang, Mingrui Li, Haiming Li, Yizhe Wang

**Affiliations:** 1 College of Marine and Environmental Sciences, Tianjin University of Science and Technology, Tianjin 300457, China; wangjiew2021@163.com; 2 State Key Laboratory of Environmental Criteria and Risk Assessment, Chinese Research Academy of Environmental Sciences, Beijing 100012, China; wzy972953@163.com (Z.W.); limingrui212@mails.ucas.ac.cn (M.L.); 3 Bayingoleng Ecological Environment Monitoring Station of Weiwu ‘er Autonomous District, Xinjiang 841000, China; mingkongqi2023@163.com; 4 Technical Centre for Soil, Agriculture and Rural Ecology and Environment, Ministry of Ecology and Environment, Beijing 100012, China; gaofugaofu@126.com

**Keywords:** coastal, sediment, pesticides, ecological risk assessment

## Abstract

Although the ecological risk of emerging contaminants is currently a research hotspot in China and abroad, few studies have investigated the ecological risk of pesticide pollutants in Chinese coastal sediments. In this study, nine pesticide pollutants included in the “List of New Key Pollutants for Control (2023 Edition)” issued by the Chinese government were used as the research objects, and the environmental exposure of pesticide pollutants in China’s coastal sediments was analyzed. The baseline sediment quality criteria were deduced using the balanced distribution method, and a multi-level ecological risk assessment of pesticides in sediment was performed. The results showed that the nine pesticide pollutants were widespread in Chinese coastal sediments, with concentrations ranging from 0.01 ng·g^−1^ to 330 ng·g^−1^. The risk quotient assessment showed that endosulfan and DDT posed medium environmental risks to the Chinese coastal sediment environment, and PCBs posed medium risks in some bays of the East China Sea. The semi-probabilistic, optimized semi-probability evaluation and joint probability curve (JPC) assessments all show that endosulfan and DDT pose a certain degree of risk to the environment.

## 1. Introduction

Persistent organic pollutants (POPs) have characteristics such as biological toxicity [1,2,3], environmental persistence [4,5], and bioaccumulation [6,7]. They are widespread in the environment and pose great risks to human health and the environment. The Chinese government has attached great importance to the control of pollutants in recent years. In December 2022, the Chinese government released the “List of New Pollutants for Key Control (2023 Edition)”. The list contains 14 chlorinated hydrocarbons including chlorinated pesticides: Chlordane, Mirex, DDTs, HCH isomers, Endosulfans, HCB, and PCBs.

Pesticide pollution is very common. For example, organochlorine and other pesticide pollutants were detected in Cirebon [8], the East China Sea [9], and Xiangshan Bay [10] in Indonesia, and the total concentration ranges were 10–120 ng·L^−1^, 183.49–1363.77 ng·L^−1^, and 2.88–34.72 ng·L^−1^, respectively. Similarly, a variety of pesticide pollutants were also found in the sediments of the Ebro River Delta [11], the Vasai River in Mumbai [12], the iSimangaliso Wetland Park in South Africa [13], and the west coast of India [14], with concentration ranges of 50.8–1912 ng·g^−1^, 597–1538 ng·g^−1^, 26.29–283 ng·g^−1^, and 0.39–21.16 ng·g^−1^, respectively. In addition, PCBs and organochlorine pesticides were found in the sediments of Shantou Bay, China, with concentrations ranging from 0.54 to 55.5 ng·g^−1^ and 2.19 to 16.9 ng·g^−1^ [15]. Organochlorine pesticides were found in the sediments of areas of North Bohai Sea, China, and concentrations of HCH and DDT in sediments ranged from below detection (<LOD) to 1964.97 ng·g^−1^ and <LOD to 86.46 ng·g^−1^, respectively [16]. PCBs were found in the Jiaojiang Estuary of the East China Sea, with concentrations ranging from 4.93 ng·g^−1^ to 108.79 ng·g^−1^ [17].

The ecological risk of pesticides in the aquatic environment is a topic of widespread concern around the world. For example, Guo et al. used the risk quotient method and the probabilistic risk assessment method to conduct an ecological risk assessment of organochlorine pesticides in the surface waters of Meiliang Bay, Gonghu Bay, and Huikou Bay in Taihu Lake [18]. The results showed that DDT, endosulfan, and hexachlorocyclohexane (HCH) presented relatively high risks. Xu et al. used the risk quotient method to assess the ecological risks of 35 pesticides in seven watersheds in China [19]. The results showed that the ecological risks of each watershed were at a potential medium level, and pesticides were the main compounds that posed risk. Hong et al. conducted an ecological risk assessment of DDT in pond sediments in the Yangtze River–Huai River region of China, and they found that there was a moderate to high ecological risk [20]. The results showed that the total content of PCBs and DDT posed a moderate ecological risk [21]. Zhao et al. conducted an ecological risk assessment of organochlorine pesticides and polychlorinated biphenyls in surface sediments of Tianjin Haihe Estuary based on sediment quality guidelines. The results showed that organochlorine pesticides and PCBs have potential ecological risks [22].

In addition, the ecological risks of pesticides in the ocean have also received widespread attention. For example, Xie et al. used the risk quotient method to assess the ecological risk of pesticides in the coastal waters of the Liaodong Peninsula in China, and the results showed that atrazine and acetochlor had higher risks to aquatic organisms than other pesticides [23]. Wang et al. conducted an ecological risk assessment of organochlorine pesticides in the waters of Hangzhou Bay, China, and the results showed that the potential danger of organochlorine pesticides in sediments was worrying [24]. The above-mentioned studies mainly conducted ecological risk assessments based on acute toxicity data, the assessment methods were not uniform, and the areas were relatively scattered [25]. There have been few systematic studies on the ecological risk assessment of pesticides in sediments in China’s coastal waters.

Based on previous research, this study (1) analyzed the environmental exposure of pesticides in China’s coastal sediments; and (2) conducted a multi-level ecological risk assessment of pesticides in sediments based on the sediment quality criteria derived using the phase equilibrium distribution method to provide a reference for the environmental management of pollutants in China.

## 2. Materials and Methods

### 2.1. Evaluation and Selection of Data

#### 2.1.1. Environmental Concentration Data of Pollutants

The nine pesticides listed in the “List of New Pollutants for Key Control (2023 Edition)” released by China, namely chlordane, mirex, hexachlorobenzene, DDT, α-HCH, β-HCH, lindane, endosulfan technical and its related isomers, and PCBs, were used as research objects; the keywords “sediment”, “risk assessment”, “pollutants”, and “China” were used to search the Web of Science and China National Knowledge Infrastructure; and we screened them from the obtained literature on the exposure data of pesticide pollutants in China’s coastal waters. A total of 3379 exposure data points from 47 documents were collected. The detection time was from January 1998 to April 2023. The survey areas were the Bohai Sea, East China Sea, Yellow Sea, and South China Sea in China’s coastal waters. The survey areas included the key sea areas of China’s CEC control, such as the Yangtze River Estuary, the Pearl River Estuary, and Hangzhou Bay. If the data collected in this study contained values greater than the method detection limit, the actual measured values were recorded. If the monitoring data were displayed in graphic form, the average concentrations were recorded. Given the number of studies in the literature, the mean concentration for a location was calculated using measured values if greater than the method detection limits (MDL), the 1/2 MDL if < MDL, or 0 if not detected.

#### 2.1.2. Environmental Toxicity Information

According to the principles of the accuracy, relevance, and reliability of pollutant toxicity data proposed by the US Environmental Protection Agency [26], Klimmisch [27], Durda [28], Hobbs [29], and Moermond [30], the toxic effect data for nine pesticides were searched in the ECOTOX database (https://cfpub.epa.gov/ecotox/search.cfm, accessed on 7 April 2022). During the toxicity data screening process, the data quality was evaluated from the following aspects: (1) experimental design, including the testing method, experimental process, and the validity and quality control of the experimental results; (2) reagent purity (>90%); (3) the source of the tested organisms; (4) exposure conditions, including the applicability of the test system to the test substance and the test organism, the test concentration interval, exposure time, and biomass loading; and (5) data analysis, including the statistical methods and concentration response curves. Toxicity data were used for classification. In principle, the most sensitive effect indicators were selected, and the chronic toxicity data of no observed effect concentration (NOEC) or 10% effect concentration (EC10) were preferred, followed by the lowest observed effect concentration (LOEC) or the median effect concentration (EC50) [31].

### 2.2. Derivation of Sediment Quality Benchmarks Using the Phase Equilibrium Distribution Method

There are many methods for deriving sediment benchmarks. For non-ionic organic compounds, the US Environmental Protection Agency recommends derivation using the equilibrium distribution method [32]. This method assumes that benthic and overlying aquatic organisms have the same sensitivity to the same pollutant. When the pollutants in interstitial water reach the water quality criteria (WQC), the content of pollutants in the sediment is the sediment quality criteria (SQC). The sediment quality benchmark calculation formula is as follows [32]:

K_p_ = f_oc_ × K_oc_, (1)

SQC = K_p_ × WQC, (2)

where f_oc_ is the organic carbon content, dimensionless; the K_oc_ organic carbon partition coefficient can be deduced from the octanol/water partition coefficient K_ow_, and the unit is L·kg^−1^; K_p_ is the partition coefficient of pollutants between the sediment phase and the interstitial water phase, dimensionless; WQC is the reference value of water quality, ng·L^−1^; and SQC is the sediment quality standard value, ng·g^−1^.

### 2.3. Ecological Risk Assessment

Multi-level ecological risk assessment (MLERA) is a method for the comprehensive ecological risk assessment of pollutants from a low level to a high level. This study performed an MLERA based on the ecotoxicology risk assessment framework [33,34], the risk assessment technical guidance document [35], the NORMAN priority framework for substances, and previous studies [36,37].

#### 2.3.1. First Level: Quotient Value Method

The quotient value method is the most commonly used and extensive risk assessment method. The calculation method of the risk quotient is the ratio of the average concentration of a single chemical in the sediment to the predicted no-effect concentration (PNEC). The formula is as follows [38]:

RQ = C/PNEC_sediment_, (3)

In the formula, RQ is the risk quotient, dimensionless; C is the average concentration calculated from the measured value set of a single chemical, ng·g^−1^; and PNEC_sediment_ is the predicted no-effect concentration derived by the most sensitive toxicity data with assessment factors (Afs) of 10, 20, or 100 depending on test endpoints of NOEC or EC10, LOEC, or EC50 [31,39].

When the RQ is less than 0.1, it is considered that there is no risk; when 0.1 ≤ RQ < 1, the pollutant is considered to have low risk; when 1 ≤ RQ < 10, the pollutant is considered to have medium risk; when RQ ≥ 10, it is considered that the pollutant has a high risk [31,40]. Although the quotient value method can initially reflect the relative risk of a pollutant, it cannot explain the actual impact of the pollutant on aquatic organisms.

#### 2.3.2. Second Level: Semi-Probability Method

The semi-probability method compares the measured ambient concentration of an individual chemical at each sampling point with its PNEC value. Pollution concentrations above the PNEC pose a potential risk to aquatic organisms, while concentrations below the PNEC are considered to pose an insignificant risk. Therefore, frequencies exceeding the PNEC can be used to prioritize pollutants. The frequency at which a target chemical exceeds the PNEC (F) can be calculated as the number of sampling points whose concentration exceeds the PNEC divided by the total number of sampling points. The results reveal the proportion of sites showing potential risk potential. The formula is as follows [38]:

F = n/N × 100%,(4)

where F is the frequency exceeding PNEC, dimensionless; n is the number of sampling stations whose concentration exceeds the PNEC; and N is the total number of stations [41].

#### 2.3.3. Third Level: Semi-Probability Method for Optimization

The current RQ based on the average concentration in water may be biased by the detection frequency. When screening high-risk compounds, it is a trend to consider both the concentration and frequency, so the optimal level of risk assessment can be performed according to the NORMAN network [37,42,43,44]. The product of the RQ_max_ value and the frequency of PNEC exceeding the standard is the priority index (PI), which can more clearly show the pesticide pollutants that should be focused on in China’s coastal sediments. The formula is as follows [38]:

PI = RQ_max_ × F, (5)

In the formula, PI is the priority index, dimensionless; RQ_max_ is the risk quotient calculated based on the maximum concentration, dimensionless; and F is the frequency at which the concentration exceeds the PNEC, dimensionless.

When the PI is less than 1, the risk of the pollutant is low and the pollutant does not pose an ecological risk to the environment.

#### 2.3.4. Fourth Level: Joint Probability Curves

There are many subjective factors in the process of formulating PNECs, which are determined based on the effects of small concentrations reported by a limited number of studies, and the results may not be repeatable. Joint probability curve methods can remedy this deficiency through using the linear regression of two datasets to calculate the probability that a concentration will adversely affect a specific proportion (%) of a species, and classifying the risk as minimal, low, medium, or high [45,46,47]. The formula is as follows [38]:

Risk product = exceedance probability × magnitude of effect,(6)

Risk < 0.25% is classified as minimal risk; risk ≥ 0.25% and <2% is classified as low risk; risk ≥ 2% and <10% is classified as medium risk; and risk ≥ 10% is classified as high risk.

## 3. Results and Discussion

### 3.1. Distribution of Pesticides in China’s Coastal Sediments

The nine pesticides in this study were from the “List of Emerging Contaminants for Key Control“. Nine pesticide pollutants with relevant research data in China’s coastal sediments were used as research objects to evaluate their risk levels in China’s coastal sediments.

**Figure 1:**
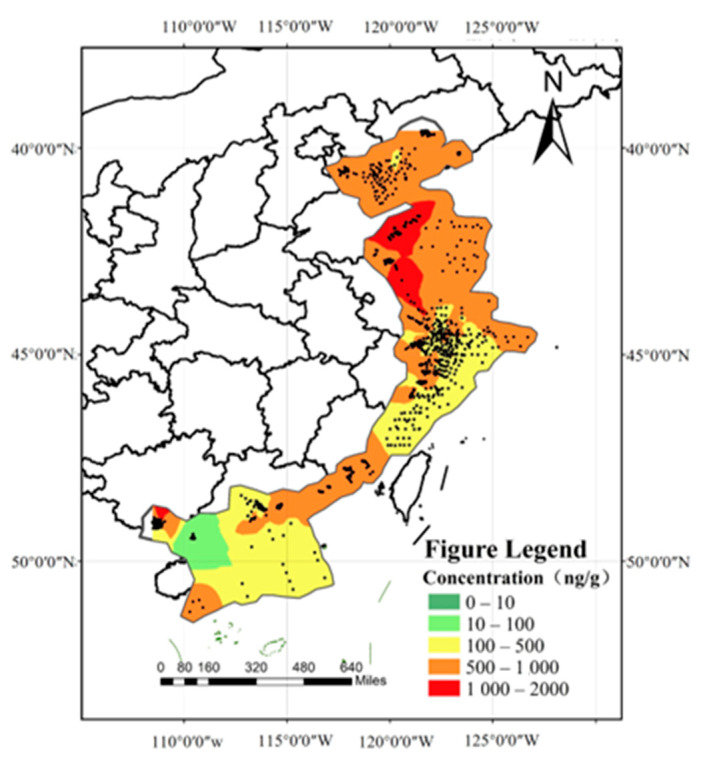
Concentration gradient map of pesticide pollutants in China’s coastal waters.

**Figure 2:**
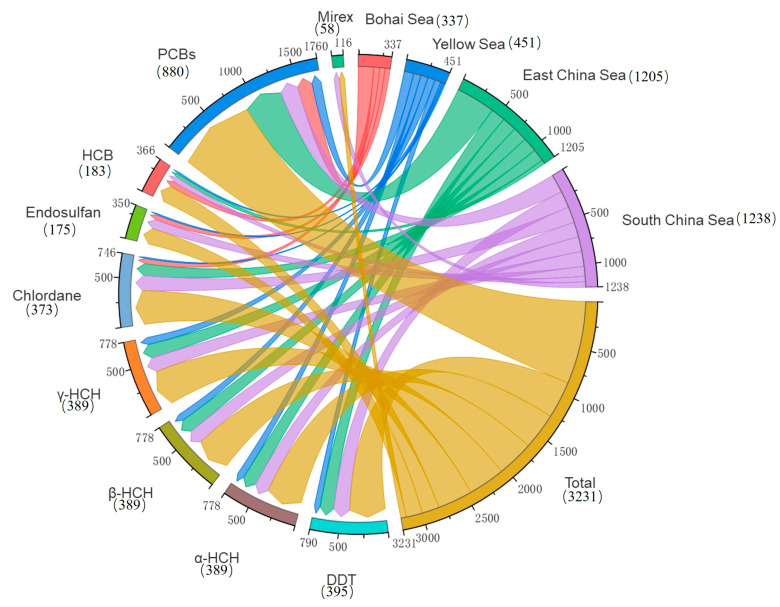
Total number of pesticide pollutants in China’s coastal sediments.

**Figure 3:**
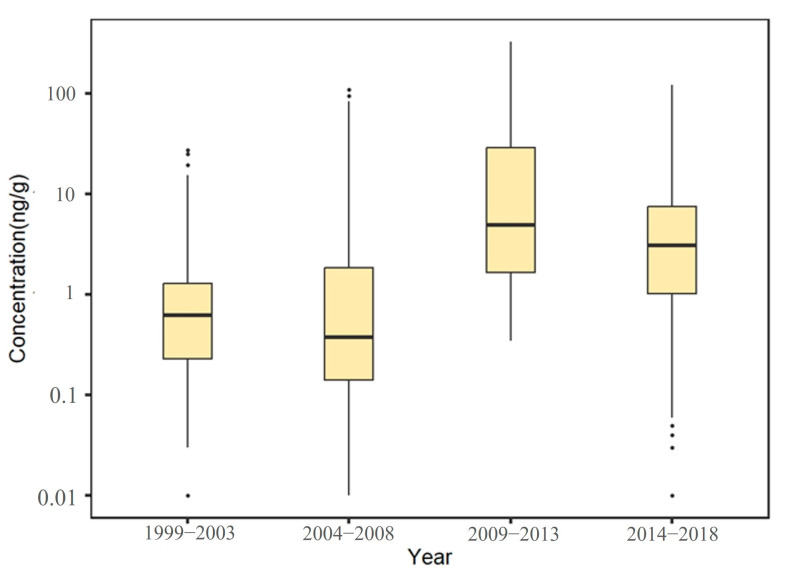
Temporal variation trends of pesticide pollutant concentrations in coastal sediments of China.

**Figure 4:**
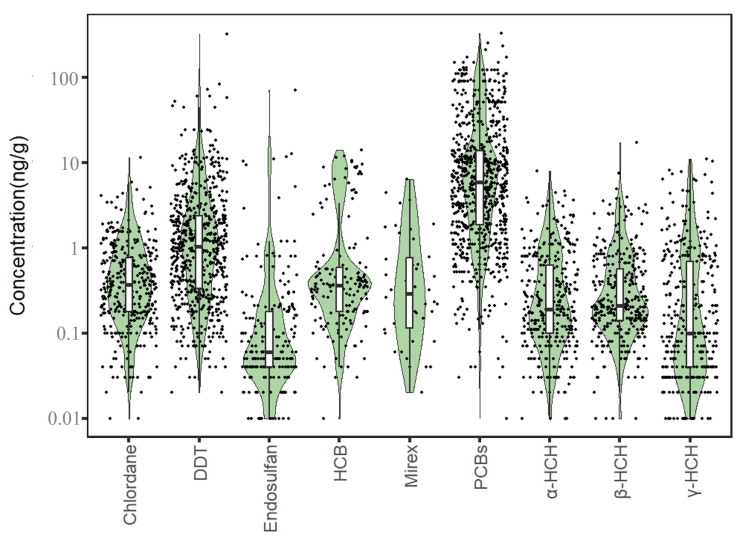
Concentrations of pesticide pollutants in the offshore sediments of total sea areas in China.

A total of 3290 exposure data points of nine target chemicals were collected in the coastal waters of China, distributed in the Bohai Sea, Yellow Sea, East China Sea, and South China Sea (Figure 1). Among them, the South China Sea had the most types of pesticide pollutants (nine types), followed by the Yellow Sea (eight types), the East China Sea (seven types), and the Bohai Sea, which had had the fewest types of pesticides (four types) (Figure 2). As shown in Figure 1, the concentration of pollutants in the Yellow Sea was the highest, and the concentration of pollutants in most sea areas ranged from 500 to 1000 ng·g^−1^ dw, with the highest concentration reaching 1188 ng·g^−1^. It can be seen from Figure 3 that over time, the concentrations of organochlorine pesticides measured in China’s coastal sediments show an increasing trend. During the five-year period from 2009 to 2013, the highest exposure concentrations of organochlorine pesticides and PCBs were measured in coastal sediments in China. Overall, the pollution of organochlorine pesticides and PCBs in China’s coastal sediments is intensifying. This finding showed that there were more pesticide residues in this sea area. In addition, the concentration of pollutants in the South China Sea was relatively high. The concentration of pollutants in most sea areas was between 1 and 10 ng·g^−1^ dw, and the concentration of pollutants in some coastal areas was between 100 and 500 ng·g^−1^ dw. It can be seen from Figure 2 that the most frequently reported sea area is the South China Sea, with a total of 1238 samples reported, followed by the East China Sea, with a total of 1205 samples reported. The main reason for this is that the Yangtze River Estuary and the Pearl River Estuary are national key sea areas, and there are many research studies on pesticide pollutants. Figure 4 shows the environmental concentrations of the nine compounds. Across the country, except for mirex, other pesticide pollutants were detected at a relatively high frequency (75–100%) in China’s coastal sediments. The most detected pollutant was polychlorinated biphenyls, with 880 concentration data points, which were detected in the Bohai Sea, Yellow Sea, East China Sea, and South China Sea. The pollutants α-HCH, β-HCH, and lindane, as HCH isomers, were tested the same number of times. It is worth noting that the research on some pesticide pollutants is relatively limited. For example, mirex was only reported in the South China Sea, and only 58 samples were reported. Considering its detection frequency of 72%, further research is needed.

### 3.2. Toxic Effects of Pesticide Pollutants

In this study, available chronic toxicity data on aquatic organisms were collected for nine pesticide-type pollutants (Table 1). The results shown in Table 1 represent the most sensitive endpoints for the nine pesticides. The data obtained in this study contained individual toxicity data for nine species. Among them, there were four vertebrates, four invertebrates, and one primary producer. The threshold range of chronic toxicity endpoints in vertebrates is 0.1–32,000 ng·L^−1^, and the threshold range of invertebrates is 1–65,000 ng·L^−1^. The chronic toxicity endpoint for the primary producer is 12,000 ng·L^−1^. Among the nine pesticides, endosulfan, chlordane, and DDT were extremely toxic to organisms, with chronic toxicity endpoints of 0.1 ng·L^−1^, 1 ng·L^−1^, and 5 ng·L^−1^, respectively.

### 3.3. Risk Characterizations

#### 3.3.1. First Level: Quotient Value Method

**Figure 5:**
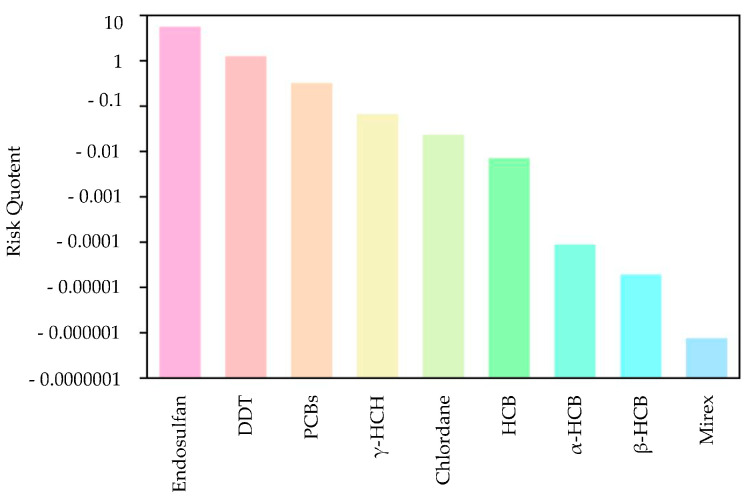
Sorting chart of the risk values of nine pesticide pollutants.

**Figure 6:**
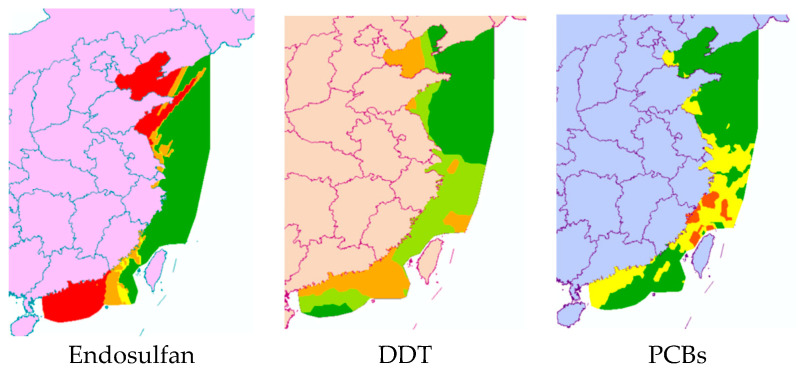
Risk map of three pesticide pollutants in China’s coastal sediments.

First, the quotient value method was used to conduct the first-level risk assessment of nine chlorinated hydrocarbon pollutants. Figure 5 shows the ranking of the risk values of nine chlorinated hydrocarbon pollutants, from high to low: endosulfan, DDT, PCBs, lindane, chlordane, HCB, α-HCH, β-HCH, and mirex. Among them, the RQ values of endosulfan and DDT were between 1 and 10, indicating that endosulfan and DDT had moderate environmental risks, and the other seven pesticide pollutants had low risks. Figure 6 further shows the risk map of the top three pollutants, namely endosulfan, DDT, and PCBs, in coastal sediments in different sea areas of China. It can be seen from Figure 6 that when evaluating each sea area separately, endosulfan has a relatively high risk in the Bohai Sea and the South China Sea; DDT poses a medium risk around the Bohai Sea and the South China Sea; and PCBs have low risks near the East China Sea and South China Sea, with moderate risks in some bays of the East China Sea.

#### 3.3.2. Second Level: Semi-Probability Method

Using the average concentration to assess the risk of pollutants produces a large error, and it is impossible to accurately assess the actual exposure of pollutants in each area. Therefore, the semi-probability method was used for the second-level risk assessment to quantify the probability that the exposure concentrations of nine chlorinated hydrocarbon pollutants in China’s coastal sediments would exceed the PNEC of aquatic organisms, and the percentage exceeding the PNEC value was identified. The results showed that the four chlorinated hydrocarbon pollutants of endosulfan, DDT, PCBs, and lindane had environmental risks, and they had adverse effects on some sensitive species, while the frequencies of other pollutants exceeding the PNEC were all zero and the environmental risks were low. Among them, endosulfan had the highest probability of exceeding the PNEC at 71%, followed by DDT at 25%, while lindane and PCBs each had several points where the exposure concentration exceeded the PNEC value.

The comparison of the two methods showed that the risk of the three chlorinated hydrocarbon pollutants (endosulfan, DDT, and PCBs) to the environment could be observed using the quotient value method and the semi-probability method, but the quotient value method could not evaluate the risk of lindane. For environmental risks, the semi-probability method could further accurately detect the excess of lindane at individual points and provide technical support for the next step of precise management.

#### 3.3.3. Third Level: Semi-Probability Method for Optimization

The optimized semi-probability method was used to conduct the third-level risk assessment. The prioritization indexes (PIs) of the four chlorinated hydrocarbon pollutants of endosulfan, DDT, PCBs, and lindane were calculated, and risk characterization was performed. The PIs of four chlorinated hydrocarbon pollutants were endosulfan at a PI of 383, DDT at a PI of 29.7, PCBs at a PI of 2.6, and lindane at a PI of 3.1 × 10^−2^. The results showed that the PI values of endosulfan, DDT, and PCBs were all greater than 1, and therefore posed risks to the environment, whereas the PI value of lindane was less than 1, and the risk to the environment was low. It is suggested that endosulfan and DDT be treated as the priority compounds in China’s coastal sediments for key research.

The comparison revealed that for the four pesticide pollutants, although the quotient value method judged that both endosulfan and DDT had moderate risks, the difference in PI between the two obtained using the optimized semi-probability method was nearly 13 times, the main reason being that for endosulfan, the PNEC was exceeded more frequently (71%). In addition, although the commercial value method determined that lindane had no risk to the environment, and the semi-probability method indicated that lindane posed a certain risk to the environment, the evaluation using the optimized semi-probability method showed that the risk of lindane was low and it did not pose a risk to the environment. This result was attributed to the low frequency of PNEC exceedance in lindane (0.5%).

#### 3.3.4. Joint Probability Curve Method

**Figure 7:**
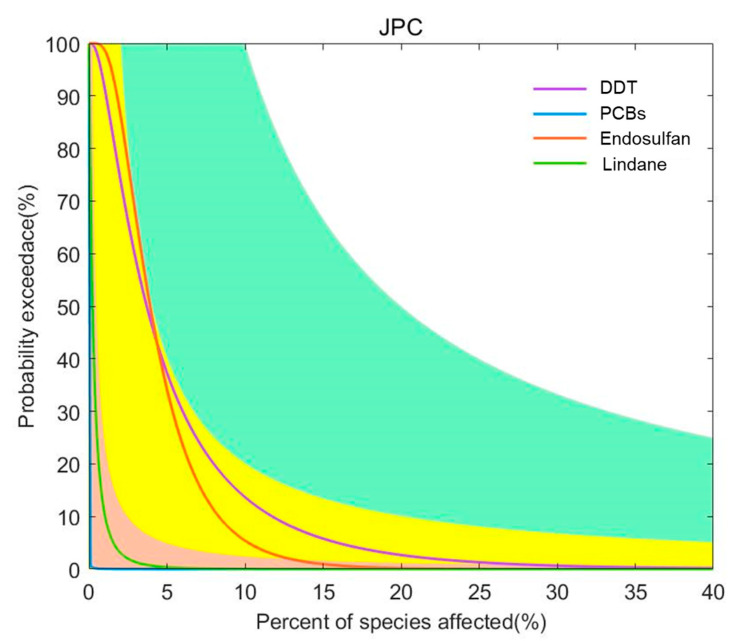
Joint probability curve of the toxicity of pesticide pollutants in the coastal waters of China. Risk classification: orange: minimum risk; yellow: low risk; green: medium risk; white: high risk.

For the four pesticide pollutants of endosulfan, DDT, PCBs, and lindane, the joint probability curve method was used to conduct a sophisticated fourth-level risk assessment based on ecotoxicity data. The joint probability curves for each pollutant were obtained by integrating the impact of China’s coastal sediment concentration distribution on the chronic toxicity data of different species, and they were used to indicate the probability of exceeding different levels of impact (Figure 7). As can be seen from Figure 7, endosulfan and DDT have a low risk of chronic effects on aquatic organisms.

#### 3.3.5. Comparing the Risk Assessment Results Generated Using the Four Methods

In this paper, four methods were used to conduct an MLERA of pesticide pollutants in China’s coastal sediments. The quotient value method is often used in most quantitative or semi-quantitative ecological risk assessments. However, this method is usually conservative in determining exposure and selecting toxicity reference values; it provides only a rough estimate of risk, and there are many uncertainties in the calculation. Moreover, the quotient value method does not take into account the differences in the exposure of individuals within the population and the differences in the chronic effects of exposed species. The semi-probability method and the optimized semi-probability method can express the probability of ecological risks posed by chemicals to aquatic organisms in the coastal areas of the country. This method conducts ecological risk assessment based mainly on the probability that the detected concentration of pollutants will exceed the PNEC. The optimized semi-probability method considers both the environmental concentration and frequency, and it evaluates the ecological risk of regional pollutants based on the actual situation, which is beneficial to the management of regional pollutants. For example, after using the semi-probability method to assess the ecological risk of lindane, it was found that the environmental concentration of lindane exceeded the PNEC value at some points, while the risk of lindane determined using the optimized semi-probability method indicated that lindane posed no significant risk (PI = 3.1 × 10^−2^). However, lindane still has points exceeding the PNEC in China’s coastal sediments, especially in waters where lindane poses a potential risk to aquatic organisms, and its risk should not be completely ignored. The main disadvantage of the optimized semi-probability method is that in this study, the toxicity data of the most sensitive species were used as the PNEC value, and the sediment reference value was derived using the phase equilibrium distribution method, which did not take into account the range of species present in the environment. Therefore, the predicted risk needs to be confirmed using the joint probability curve. Taking endosulfan as an example, the frequency of the PNEC exceeding the standard was 71%, and the PI was 383, but the results of the joint probability curve showed that its relative risk in sea areas was relatively low.

## 4. Conclusions

This study analyzed the environmental exposure of pesticide pollutants in China’s offshore sediments and conducted a multi-level ecological risk assessment of pesticides in sediments. The results show the following:

(1) Nine kinds of pesticide pollutants are widespread in China’s coastal sediments, with concentrations ranging from 0.01 ng·L^−1^ to 330 ng·L^−1^.
(2) The first-level quotient method assessment showed that the risk quotients of the nine chlorinated hydrocarbon pollutants were ranked in descending order as endosulfan, DDT, PCBs, lindane, chlordane, HCB, α-HCH, β-HCH, and mirex. Among these pollutants, endosulfan and DDT posed a medium environmental risk to the sediments in the coastal waters of China, and PCBs posed a medium risk in some bays of the East China Sea. The second-level semi-probability assessment showed that endosulfan, DDT, lindane, and PCBs should be considered as priority pollutants. The semi-probability evaluation results of the third-level optimization show that the focus should be on the ecological risks of endosulfan, DDT, PCBs, and lindane in China’s coastal sediments, especially endosulfan and DDT. The four-level joint probability curve method assessment shows that endosulfan and DDT have a low risk of chronic effects on aquatic organisms.
(3) Based on the four evaluation methods, this study concludes that endosulfan and DDT pose environmental risks to China’s coastal sediments.

## Figures and Tables

**Table 1:** Toxicity of nine pesticides to aquatic organisms.

Chemicals	Endpoint	Concentration (ng·L^−1^)	AF	WQC	PNEC_sediment_
DDT	NOEC	5	10	0.5	4.9
β-HCH	NOEC	32,000	10	3200	27,646
HCB	NOEC	96.6	10	9.66	193
α-HCH	EC50	65,000	100	650	5616
Lindane	NOEC	10	10	1	8.6
Endosulfan	NOEC	0.1	10	0.01	0.03
Chlordane	LOEC	1	20	0.05	27.5
PCBs	LOEC	15	20	0.75	58.5
Mirex	EC50	12,000	100	120	841,820

Note: Toxicological data of the nine pesticides were obtained from the ECOTOX knowledge base (https://cfpub.epa.gov/ecotox/search.cfm, accessed on 7 April 2022). PNEC: predicted no-effect concentration.

## Data Availability

Data are contained within the article.
